# Sustainability of the Olive Oil System

**DOI:** 10.3390/foods10081730

**Published:** 2021-07-27

**Authors:** Cristina Alamprese, Francesco Caponio, Emma Chiavaro

**Affiliations:** 1 Department of Food, Environmental, Nutritional Sciences (DeFENS), Università degli Studi di Milano, via G. Celoria 2, 20133 Milan, Italy; 2 Department of Soil, Plant and Food Science (DISSPA), Università degli Studi di Bari Aldo Moro, via Amendola, 165/A, 70126 Bari, Italy; francesco.caponio@uniba.it; 3 Department of Food and Drug, University of Parma, Parco Area delle Scienze 27/A, 43124 Parma, Italy; emma.chiavaro@unipr.it

Sustainability is a widely accepted goal across many sectors of our society and, according to new concepts, it includes resilience and adaptive capacity. Resilience is important for a system to guarantee the maintenance of functions and structures, including when subjected to shocks. Adaptability is fundamental to face unpredictability and unforeseen changes. Both aspects must be considered in the sustainable development of the olive oil system, including environmental, economic, and social issues, but also the improvement of the well-established historical tradition by maximizing the process efficiency and the quality of the end product. Therefore, this Special Issue about “Sustainability of Olive Oil System” intends to give an overview of several aspects related to sustainability of the olive oil processing chain, in order to open minds to new “sustainable thinking”.

Starting from olive production, sustainability can be improved by using chemical-free alternatives to pesticides and organic production. Rotondi et al. [1] explored the effectiveness of kaolin-based and zeolitite-based particle films for hindering the attacks of the olive fruit fly (*Bactrocera oleae*), evaluating leaf gas exchanges and leaf optical properties. The zeolitite-based film showed the best performance, exerting a protective effect against olive fruit fly attacks without altering the leaf gas exchanges. Moreover, olive oils obtained from zeolitite-based particle film treatment showed intensities of gustatory and olfactory pleasant flavors higher than those of oils produced from kaolin and untreated olives. Carrapiso et al. [2] evaluated the effects on virgin olive oil characteristics of organic production without irrigation, traditional harvesting methods (tree vs. ground picked fruits), and harvesting time (over a six-week period). Organic production affected physical-chemical parameters and volatile compounds less than the harvesting method. Otherwise, a higher content in total phenols was found in the organic oils than in the conventional ones, probably explaining the increase in oil stability and the differences in the volatile compounds.

The valorization of minor olive accessions could represent a good way to improve the qualitative production of a specific territory while protecting biodiversity, an important aspect of sustainability. Four minor Italian cultivars were exploited by Piscopo et al. [3] to improve extra virgin olive oil (EVOO) production in the Calabria region; they obtained in most cases good quality oil in terms of free acidity, peroxides, spectrophotometric indexes, fatty acid composition, and bioactive compounds. Squeo et al. [4] investigated the cultivar “Oliva Rossa”, which represents an old landrace belonging to the autochthon Apulian olive germplasm. The authors showed that the extracted virgin olive oils had a medium to high level of oleic acid. With colder temperatures, a higher content of monounsaturated fatty acids and antioxidants was observed, as well as a higher oleic/linoleic ratio. The phenolic profile was dominated by secoiridoid derivatives, which might indicate a product with remarkable pungent and bitter notes. Similarly, the volatile profile was dominated by the compounds arising from the lipoxygenase pathway. The recovery and valorization of other minor Italian olive cultivars were further investigated by Sabetta et al. [5]. A pattern of nine minor genotypes cultivated in three Italian regions was molecularly fingerprinted with 12 nuclear microsatellites that were able to unequivocally identify all genotypes. In addition, the monovarietal oils were evaluated for the principal phenolic compounds and the expression levels of related genes at different fruit development stages were investigated.

An important key factor to address emerging challenges of sustainable food consumption is the reduction of the environmental footprint of packed food. Thus, the performance of two innovative packaging materials in protecting EVOO from oxidation phenomena was investigated by Farris et al. [6]. In particular, a transparent plastic film loaded with a UV-blocker and a metallized material were compared to brown-amber glass during accelerated shelf-life tests at 40 and 60 °C. The transparent film emerged as the best-performing material to preserve EVOO quality.

A key role in sustainability is played by green chemistry, which can provide online techniques for automatic evaluation of food quality and optimization of food processes, while minimizing the use of hazardous materials. In this context, fingerprint techniques are valuable tools for both quality assessment and authentication issues. Grassi et al. [7] demonstrated that near-infrared (NIR) spectroscopy can be used in the field or at the mill entrance for a quick classification of the intact olive drupes as a function of their chemical parameters (moisture, oil content, soluble solids, total phenolic content, and antioxidant activity), in order to better design the olive oil quality. Lia et al. [8] developed a nuclear magnetic resonance (NMR) method for the discrimination of Maltese and non-Maltese EVOO, showing a higher effectiveness of ^13^C NMR rather than ^1^H NMR. Physical and thermal analyses were proposed by Paciulli et al. [9] as fast and green techniques to identify botanical and geographical origin of EVOO. In particular, thirteen EVOO samples obtained from minor olive cultivars, harvested at three different ripening stages in four Italian regions (Abruzzo, Apulia, Sardinia, and Calabria), were investigated for thermal properties, viscosity, and color, as influenced by fatty acid composition and chlorophyll content. The most influential thermal parameters and fatty acids were used to identify possible sample clusters by means of principal component analysis; while a clear distribution of the samples based on their botanical and geographical origin was evident, no pattern was highlighted in terms of olive harvesting time. Moreover, Paradiso et al. [10] proposed a green method for the determination of hydroxytyrosol and tyrosol content in EVOO, based on the use of a natural deep eutectic solvent composed of lactic acid and glucose for the liquid/liquid extraction step, followed by UV-spectrophotometric analysis.

Maximization of the production process efficiency goes through the valorization of olive oil by-products, by using polyphenolic extracts derived from olive leaves and mill wastewater as food ingredients. The papers by Flamminii et al. [11] and Conte et al. [12] suggest the use of free or encapsulated polyphenolic extracts in mayonnaise and gluten-free breadsticks, demonstrating the possibility of developing healthy foods, with extended shelf life. The likelihood of consumers’ acceptance of these kinds of foods obtained with upcycled ingredients of olive oil production was studied by Perito et al. [13]. The authors found that, despite the negative influence of food technophobia, a core of sustainability-minded consumers interested in organic or local products could also favor the uptake of these foods enriched with ingredients made from olive oil by-products. Development of organic or local food products with upcycled ingredients could potentially be the right way to increase the probability of consumers’ acceptance. Indeed, Carzedda et al. [14] investigated Italian consumers’ behavior towards EVOO organic production methods and geographical origin to quantify the willingness to pay for these two attributes. Findings showed positive preference for origin attributes, especially linked to local productions.

The olive oil by-products are attracting great interest also in the pharmaceutical and renewable energy fields. Posadino et al. [15] produced a phenolic-rich extract from olive mill wastewater to assess the protection against oxidative cell death in human vascular cells. The tested extract protected cells from oxidative stress-induced cell death, failing indeed to interfere with cell viability and even with the metabolism, except for the highest tested concentration. Centrone et al. [16] explored the biological actions of extracts deriving from different olive by-products, including olive pomace, olive wastewater, and olive leaf, on human colorectal carcinoma HCT8 cells. Different effects on reactive oxygen species’ generation and cell viability were found: the extract obtained from the olive mill wastewater showed higher antioxidant ability compared with the extracts derived from olive pomace and olive leaves. These biological effects may be related to the different phenolic composition of the extracts. Actually, the olive mill wastewater extract contained the highest amounts of hydroxytyrosol and tyrosol, which are considered potent antioxidant compounds. The advantages of using farm and food industry by-products to produce renewable energy as well as organic fertilizers, which could be used in situ to enhance farm sustainability, were demonstrated by Benalia et al. [17]. The authors explored the anaerobic co-digestion of olive mill wastewater to produce biogas and biomethane. Different mixtures of olive mill wastewater were tested under mesophilic conditions. By applying the life-cycle assessment (LCA) approach, it was demonstrated that a good biogas ecoprofile and a high process profitability can be obtained using 20% (*v*/*v*) olive mill wastewater. 

Life-cycle-based methodologies are very powerful and reliable tools to quantify the impact generated from a product/service along the entire production process and throughout its whole duration. Nikkhah et al. [18] applied LCA to measure the circularity of refining oil from olive kernel, a common source of waste in olive fruit processing systems. The authors reported that the global warming potential of 1 kg oil produced from olive kernel was 1.37 kg CO_2_eq, while the calculated damage of 1 kg oil production to human health, ecosystem quality, and resource depletion was 5.29 × 10^−7^ disability-adjusted life years (DALY), 0.12 PDF m^2^ year., and 24.40 MJ, respectively. Pampuri et al. [19] quantified the environmental impact of four lab-scale food preparations (vegan mayonnaise, salad dressing, biscuits, and gluten-free breadsticks) enriched with phenolic extracts from olive oil by-products (i.e., mill wastewater and olive leaves), considering technological and nutritional parameters. The authors concluded that the phenolic extraction and encapsulation, even if characterized by low production yields, energy-intensive operations, and the partial use of chemical reagents, made a non-negligible environmental impact contribution to the food preparation. The addition of phenolic extracts to food products led to an enhanced environmental impact of the production process, but also to improved technological and nutritional performances. Impacts could be reduced through a scale-up process.

In summary, this Special Issue provides evidence that olive oil system sustainability can be improved in different ways, from the enhancement of biodiversity to the exploitation of waste and by-products for food and health-related purposes in a circular economy perspective.
